# Taxonomic Bias Persists in Ecology and Evolution Research Despite Decades of Concern

**DOI:** 10.1002/ece3.74450

**Published:** 2026-10-04

**Authors:** Pietro Pollo, Michael M. Kasumovic

**Affiliations:** ^1^ Evolution & Ecology Research Centre, School of Biological, Earth & Environmental Sciences University of New South Wales Kensington New South Wales Australia

**Keywords:** journal citation indicator, journal impact factor, knowledge imbalance, research bias, research impact, taxonomic chauvinism

## Abstract

Scientific hyperfocus on certain organisms slows innovation and hinders the generality of ecological and evolutionary inference. Yet, the extent of taxonomic bias in this research field and its potential changes over time remain poorly quantified. By assessing 1,383,803 papers and 612 journals, we show that ecology and evolution research is strongly taxonomically biased. We found that the prevalence of studies on vertebrates in the literature as a whole (but not within some pockets of ecology and evolution) has remained similar since the early 2000s, when the first studies of taxonomic bias were published. We also observed that studies on vertebrates, relative to those on invertebrates, were more prevalent in higher impact journals and, within journals, received more citations and mentions in the media. However, classifying animals into more specific groups led to context‐dependent temporal and attention patterns. We propose a series of urgent targeted actions to ameliorate taxonomic imbalances in ecology and evolution.

## Graphical abstract

Ecology and evolution research shows strong and persistent taxonomic bias, with vertebrates increasingly dominating the literature over time. By analysing 1.38 million papers across 612 journals, we find that vertebrate studies appear more often in high‐impact journals and receive disproportionately greater citation and media attention. These patterns vary when taxa are classified more finely, highlighting the need for targeted actions to correct taxonomic imbalance in the field.

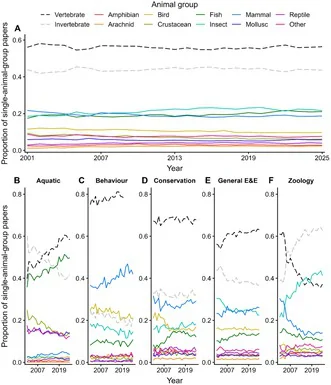

## 1 Introduction

More than 1.5 million animal species have been described to date (Bánki et al. 2026), with many more yet to be discovered (Mora et al. 2011; Moura and Jetz 2021; Stork 2018; Wiens 2023). Despite this enormous diversity, analyses of awarded grants (Leather 2009), biodiversity databases (García‐Roselló et al. 2023; Troudet et al. 2017), conservation or reintroduction projects (Adamo et al. 2022; Guénard et al. 2025; Mammola et al. 2020; Seddon et al. 2005), scientific articles (Bonnet et al. 2002; Clark and May 2002; Donaldson et al. 2017; Leather 2009; Mizuno et al. 2025; Pollo et al. 2024, 2025; Rosenthal et al. 2017; Stahlschmidt 2011; Titley et al. 2017; Zuk et al. 2014), and textbooks (Gangwani and Landin 2018) have identified that certain taxa receive disproportionate attention in the field of ecology and evolution relative to their diversity. For example, vertebrates, especially birds and mammals, often concentrate conservation (Adamo et al. 2022; Guénard et al. 2025; Mammola et al. 2020; Seddon et al. 2005) and research (Mizuno et al. 2025; Pollo et al. 2024, 2025; Rosenthal et al. 2017; Stahlschmidt 2011) efforts even though they represent only a minute portion of existing animals (Bánki et al. 2026). This inequity, called taxonomic bias or taxonomic chauvinism (Bonnet et al. 2002), can stifle innovation in medicine and engineering by limiting biomimetics (Broeckhoven and du Plessis 2022; Stuart‐Fox et al. 2023) and curb scientific advancement by impeding theoretical generality (Bonnet et al. 2002; Dochtermann et al. 2026). However, it is unclear whether taxonomic patterns in the ecology and evolution literature are universal as previous studies on taxonomic bias relied on small samples constrained by manual taxonomic identification and limited journal (Clark and May 2002; Rosenthal et al. 2017) or topic (Stahlschmidt 2011; Zuk et al. 2014) coverage. Most importantly, whether these patterns changed since taxonomic bias was first discussed by researchers in the early 2000s (Bonnet et al. 2002; Clark and May 2002) remains unknown.

Here, we explored several dimensions of taxonomic bias in the literature of ecology and evolution (612 journals and 1,383,803 papers), primarily focusing on animals. We aimed to quantify the extent of taxonomic bias and assess whether and how it has changed over time. To do so, we first assessed patterns related to journal taxonomic specialisation. Second, we investigated the proportion of papers on distinct taxonomic groups over time. Third, we evaluated the relationship between journals' impact metrics and the proportion of papers they published on each animal group. Fourth, we examined how the attention given to individual papers within journals varied with the animal group studied. We hypothesised that taxonomic patterns have not changed over time, and that papers on vertebrates, especially birds and mammals, are not only more prevalent in journals with higher impact metrics but also receive more attention than papers on other animals within the same journal and year. If these hypotheses are true, it would mean that researchers are limiting our understanding of the world and inhibiting potential innovation, possibly to pursue metrics that maximise their own research exposure.

## 2 Material and Methods

Our methodology was described in our preregistration (Pollo and Kasumovic 2025), that is, a research plan made prior to data collection and analysis (Wagenmakers et al. 2012). We highlight the deviations from our preregistration at the end of this section (see below). We report author contributions using MeRIT guidelines (i.e., stating author initials to clarify who did what; Nakagawa et al. 2023) within this section and the CRediT statement (McNutt et al. 2018) at the end of the manuscript.

### 2.1 Journal Selection and Classification

PP used Clarivate's Journal Citation Records (hereby *JCR*) classification system to select and analyse journals for this study. Although its categories may represent different variables (e.g., habitat, taxa, topic), this classification system it is widely recognised and used by institutions and researchers to partition the field of ecology and evolution. PP specifically selected all journals that were classified as ‘behavioural sciences’, ‘biodiversity conservation’, ‘ecology’, ‘entomology’, ‘evolutionary biology’, ‘marine & freshwater biology’, ‘ornithology’ or ‘zoology’ by JCR, tallying 696 journals (note that each journal could belong to multiple categories). PP then retained only journals (1) whose Clarivate's journal impact factor (JIF) (2024) was available and (2) were also indexed by Scimago Journal Rank as ‘agricultural and biological sciences’ or ‘environmental sciences’, which resulted in the selection of 612 journals (Figure S1). Afterwards, PP collated the scope of each journal (available in their respective website) and used this information to determine journals' taxonomic focus. Note that some journals that vaguely described their taxonomic criteria (e.g., laboratory animals) or that only inadvertently excluded certain organisms because of the subject they specialised on (e.g., soil ecology journals probably do not publish research on fish but have no such explicit restrictions) were still considered taxonomically unrestricted. Moreover, journals on entomology or insect science were deemed to include all arthropods unless they explicitly mentioned focusing on particular insects (e.g., coleopterans: Coleopterists Bulletin; lepidopterans: Journal of the Lepidopterists Society, Nota Lepidologica, SHILAP Revista de Lepidopterología; orthopterans and their closely related groups: Journal of Orthoptera Research).

### 2.2 Journal Impact Metrics

PP used two journal impact metrics in the present study: the journal citation indicator (JCI) and the JIF. Both metrics are calculated by JCR and were thus extracted during the journal selection process (see subsection above). The year in which these metrics are calculated by Clarivate is referred to as the JCR year, which was 2024 for the present study. Both JCI and JIF quantify citation performance but differ in key aspects. The JCI is normalised for field, document age and document type; it uses citations from the JCR year plus previous 3 years (i.e., 2024–2021) for articles and reviews (but not other document types, e.g., letters or editorials) published in the previous 3 years (i.e., 2023–2021). By contrast, JIF is an absolute metric based on citations received in the JCR year (i.e., 2024) to all documents published in the previous 2 years (i.e., 2023–2022).

### 2.3 Literature Searches

On 29 December 2025, PP retrieved information from 1,695,614 documents by searching the International Standard Serial Number (ISSN) of the 612 journals aforementioned on Scopus (accessed through the University of New South Wales). 1,389,068 documents from 606 journals remained in the dataset after PP excluded documents that were (1) officially published in 2026, (2) duplicated or (3) had missing information (DOI, journal name, abstract, title and/or author names). PP further excluded documents that were classified by Scopus as ‘book’ (*n* = 9), ‘book chapter’ (*n* = 13), ‘editorial’ (*n* = 870), ‘erratum’ (*n* = 4159) and ‘retracted’ (*n* = 214) as these do not represent valid standard research outputs. Conversely, other documents (hereby *papers*) classified by Scopus as ‘article’ (*n* = 1,305,271), ‘conference paper’ (*n* = 17,448), ‘data paper’ (*n* = 290), ‘letter’ (*n* = 2627), ‘note’ (*n* = 4104), ‘review’ (*n* = 52,165) or ‘short survey’ (*n* = 1898) remained in the dataset, tallying 1,383,803 papers from 606 journals.

### 2.4 Altmetric Data

On 20 January 2026, PP used the Altmetric API (‘Counts Only’ version) to gather all available metrics of attention indexed by Altmetric related to the papers retrieved. However, note that only 44.4% (613,768 out of 1,383,803) of the papers in our dataset were found in the Altmetric database. We specifically used three metrics of attention collected by Altmetric in our analyses (see details below): mentions in online documents (e.g., blogs, news outlets, patents, policies, social media, Wikipedia), readers in Mendeley and Altmetric score (i.e., a weighted estimate of several attention metrics).

### 2.5 Taxonomic Classification

PP selected 10 animal groups to identify papers' taxonomic focus from their title, abstract and author keywords: (1) amphibians, (2) arachnids, (3) birds, (4) crustaceans (i.e., non‐hexapod pancrustaceans), (5) fish (i.e., non‐tetrapod vertebrates), (6) insects, (7) mammals, (8) molluscs, (9) (non‐avian) reptiles and (10) other (unnamed invertebrates). MMK and PP independently evaluated the taxonomic focus of 100 papers, resulting in eight conflicts that were resolved after discussion and that generated clear criteria to evaluate papers' taxonomic focus, as follows. First, humans (i.e., any species of the genus *Homo*) were treated as non‐animal organisms for classification purposes. Second, papers could focus on distinct animal groups by exploring interactions (e.g., predator–prey, host–parasite) between species of different groups or simply by using or collecting data related to multiple taxa. However, animal groups were only identified as being the focus of a paper when they were directly or indirectly (e.g., byproducts, images) involved in the research presented (regardless of whether the research was empirical, theoretical or a review), but not when they were simply mentioned as references (see Table S1 for examples). Third, papers needed to specify taxonomic information to the level of our taxonomic classification, meaning that vague or high‐level terms (e.g., shellfish, vertebrates, zooplankton) were ignored if no specific taxonomic terms were given. Applying these criteria, PP manually identified the animal taxa examined in 6749 papers, which were used to train (*n* = 4934) and test (*n* = 1815) an algorithm that automates this process (see below).

In order to automate the classification of papers' taxonomic focus, PP compiled a set of words associated with different taxa (hereby *taxonomic keywords*), which included part of the GBIF Backbone Taxonomy (GBIF Secretariat 2023) and a customised set of keywords. PP then developed a simple algorithm to identify papers' taxonomic focus based on taxonomic keywords detected in papers' title, abstract and author keywords. PP used 4934 papers that he inspected as a training set to manually add and adjust taxonomic keywords, improving the algorithm. The algorithm was also able to identify some combinations of keywords that would generate incorrect labels if left unchecked. For instance, spider monkey is a mammal, but the terms ‘spider’ and ‘monkey’ are taxonomic keywords that the algorithm uses to identify studies focusing on arachnids and mammals, respectively. PP thus added a step in the algorithm that corrects for these occurrences.

PP evaluated the efficacy of the algorithm mentioned above in three ways. First, PP compared the manually identified taxonomic focus of the remaining 1815 papers he inspected with the equivalent labels generated by the algorithm. PP found that the algorithm had, for each taxonomic group (Table S2), at least 97.2% accuracy (i.e., proportion of correct predictions; Metz 1978), 84.4% precision (i.e., proportion of predicted positives that are truly positive; Kent et al. 1955), 95.4% sensitivity (i.e., true positive rate, also called recall; Kent et al. 1955; Yerushalmy 1947), 96.9% specificity (i.e., true negative rate; Yerushalmy 1947), and 91.2% F1‐score (i.e., harmonic mean of precision and sensitivity; van Rijsbergen 1979). Note that the training and the test sets each contained at least three papers from each of the 605 journals in the dataset (one other journal had only one paper in the dataset, which was used in the training set), ensuring a reliable comparison. Second, PP verified the proportion of papers from taxonomically specialised journals receiving impossible taxonomic labels, such as studies published in the Journal of Mammalogy identified by the algorithm as involving only animals other than mammals. He detected these gross errors in only 0.06% of all such papers (see also Figures S2 and S3). Third, PP compared the taxonomic labels generated by the algorithm with the dataset from Stahlschmidt (2011), who manually identified a single animal taxon focused by 82 papers present in our dataset. PP found that the algorithm's taxonomic classifications matched Stahlschmidt's, except for a paper on humans (classified by Stahlschmidt as mammal). However, the algorithm also identified additional animal groups for 14 of these papers besides those identified by Stahlschmidt.

### 2.6 Statistical Analyses

PP fitted several models to analyse taxonomic patterns of journals and papers. He first conducted a linear regression with JIF as the response variable and journal taxonomic scope as the predictor variable. He then performed three Dirichlet regressions, all with the same response variable (proportions of papers involving zero, one or two or more animal groups) and random effect (journal identity), but each with a different predictor variable (year of publication, JIF or JCI).

To conduct all other analyses, that is, those involving single‐animal‐group papers published in taxonomically unrestricted journals, PP used two complementary approaches. He analysed papers from all journals combined to characterise overall taxonomic patterns in the ecology and evolution literature, but also assessed how taxonomic patterns varied across this field by examining papers within journal sets. These journals sets were largely based on the JCR classification (Figure S1), but with some modifications so each journal belonged to only one set: aquatic (journals classified by JCR as ‘marine or freshwater biology’; *N* = 76), behaviour (journals classified as ‘behavioural sciences’; *N* = 16), conservation (journals classified as ‘biodiversity conservation’ but not included in the aquatic set; *N* = 62), general ecology and evolution (remaining journals classified as ‘ecology’ or ‘evolutionary biology’; *N* = 142) and zoology (all remaining journals, mostly classified as ‘zoology’ but some as ‘entomology’; *N* = 79). Within each of these approaches, two different taxonomic levels were analysed: binary (vertebrates vs. invertebrates) and specific (10 animal groups previously mentioned).

Under the scenarios described above, PP performed logistic regressions (binary taxa) or Dirichlet regressions (specific taxa), which are ideal methods to examine response variables with two or multiple levels, respectively. In these regressions, a single predictor variable was included (year of publication, JIF or JCI) along journal identity as a random effect. PP made sensitivity analyses of these models by excluding journals with JIF > 6 and JCI > 2, considering the relationship between a journal impact metric and the prevalence of papers on an animal group as different from zero when both model versions yielded the same qualitative results (i.e., both negative or positive).

Lastly, PP fitted several generalised linear mixed models (GLMMs) with a negative binomial error distribution to explore the attention given to single‐animal‐group papers depending on the taxon they focused on. In these GLMMs, the response variable could be one of four attention metrics: the number of (1) citations by other papers according to Scopus, (2) mentions in online documents, (3) readers in Mendeley or (4) (rounded) Altmetric score. In these models, journal identity, year of publication and their interaction were random factors and either binary or specific animal groups along with JIF were predictor variables. These models, however, could bias estimates as most journals did not publish papers on all animal groups every given year, leaving several journal‐year‐taxon combinations absent from the data. Moreover, Altmetric data were more likely to be available for papers published more recently and in journals with higher impact factor (Figure S4), potentially biasing estimates given the taxonomic patterns we found related to time and journal impact metrics (see Section 3). PP therefore conducted sensitivity analyses by examining only papers published in specific years (see below) in journals that had at least one paper focusing on each of the 10 animal groups aforementioned and had an impact factor up to 6. Given that JIF was included as a predictor variable in all analyses of attention patterns, estimates were calculated for a journal with an average impact factor (i.e., approximately 2).

Our analyses involved papers published over a certain timespan. First, PP assessed all papers, regardless of when they were published, to infer general taxonomic patterns in the literature. Second, PP used papers published between 2001 and 2025 to evaluate taxonomic patterns over time as the first reports on this topic (e.g., Bonnet et al. 2002; Clark and May 2002) were published at the beginning of this period. Third, PP used papers published between 2021 and 2025 to assess recent taxonomic patterns related to journal impact metrics as these metrics could be unrepresentative of past periods (historic journal impact metrics were unavailable). Fourth, PP restricted the sensitivity analyses of attention metric patterns to papers published from 2018 to 2022 (these years had the largest numbers of papers with available Altmetric data), analysing each year separately.

PP conducted all analyses using R v. 4.5.1 (R Core Team 2025). PP fitted GLMMs using the package *glmmTMB* v. 1.1.12 (Brooks, Mollie, et al. 2017; McGillycuddy et al. 2025) and performed pairwise comparisons for these models using the packages *emmeans* v. 1.11.2.8 (Lenth 2025), *multcomp* v. 1.4.28 (Hothorn et al. 2008) and *multcompView* v. 0.1.10 (Graves et al. 2024). PP verified that the assumptions of GLMMs were met using the package *DHARMa* v. 0.4.7 (Hartig 2016). PP conducted logistic and Dirichlet regressions using Bayesian models in the package *brms* v. 2.23.0 (Bürkner 2017, 2018, 2021) and the package *cmdstanr* v. 0.9.0 (Gabry et al. 2025). For these regressions, PP ran three chains, each with 5000 iterations plus another 5000 burn‐in iterations, adopting Stan's standard uninformative priors and setting the adaptive delta to 0.9. PP then computed predictions and trends from these regressions using the package *marginaleffects* v. 0.31.0 (Arel‐Bundock et al. 2024). PP visually checked that all chains converged and verified that *R*‐hat values were lower than 1.01. Continuous variables in all models fitted were scaled by subtracting the mean from each value and then dividing the result by the standard deviation.

### 2.7 Deviations From the Preregistration

Some of our methodology was described in our preregistration (Pollo and Kasumovic 2025). However, we greatly expanded our original plan, which culminated in two separate projects: The present study describing patterns of taxonomic bias in the literature of ecology and evolution, and another study exploring the potential drivers of taxonomic bias from data collected via a survey with behavioural ecologists (Pollo and Kasumovic 2026). Changes from our preregistration are summarised as follows. First, we initially planned to examine taxonomic bias in papers published in 10 behaviour journals, but later expanded the range of our study to a much broader selection of journals related to ecology and evolution (see details below). Second, we originally planned to include non‐animal organisms when classifying papers' taxonomic focus. Nonetheless, distinguishing the use of non‐animal organisms as research objects from their use as animals' environment, substrate or food proved to be difficult. Therefore, we opted to only identify animal taxa in our study. Third, we originally planned to improve the algorithm using up to 500 papers, but used many more instead to ensure the reliability of the taxonomic classification algorithm (see details above). Fourth, many analyses in the present study, such as those involving data from Altmetric, were not initially planned in our preregistration. Fifth, none of our statistical models were formulated in the preregistration, so all our analyses can be considered ad‐hoc.

## 3 Results

### 3.1 Taxonomic Patterns Across Journals

Almost 40% (235 out of 612) of journals had explicit taxonomic restrictions, and 42.1% of these taxonomically specialised journals (99 out of 235) focused exclusively on all or some arthropods (Figure 1A). Only journals specialised on birds had, on average, a lower impact factor than taxonomically unrestricted journals or those specialised in humans or non‐animal organisms (Figure 1B). However, the small number of journals specialised in other animal groups (e.g., arachnids, molluscs) may have limited our ability to detect statistical differences between their average impact factor and that of taxonomically unrestricted journals.

**FIGURE 1:**
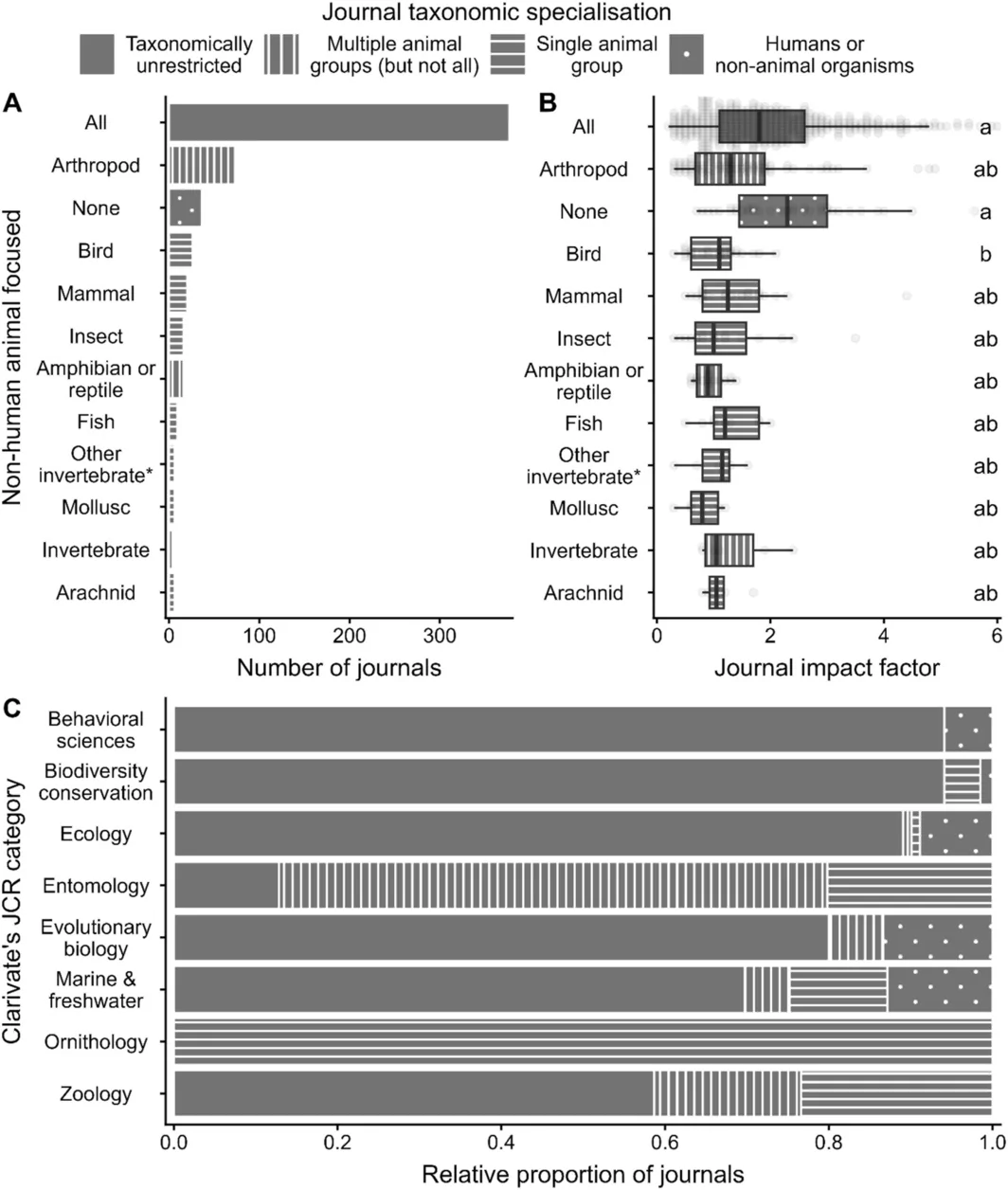
Taxonomic specialisation of ecology and evolution journals. Panel A shows the number of journals focusing on each non‐human animal taxon, with panel B showing the impact factor of these journals in 2024 (curtailed *x*‐axis with 6 as maximum value; only taxa focused by at least five journals are shown). Panel C shows the taxonomic specialisation of journals within each Journal Citation Report (JCR) category. In Panels A and B, ‘Other invertebrate*’ refers to invertebrates other than arachnids, crustaceans, insects and molluscs. In panel B, dots represent individual journals, boxes enclose 50% of the data (interquartile range), the whiskers contain values up to 1.5 times the interquartile range, the solid line within the boxes represents the median, and distinct letters represent statistical differences between groups of journals with different focused taxa (*z*‐values with *p* < 0.05 for all pairwise comparisons).

Following Clarivate's Journal Citation Report (JCR; Figure S1) classification system, we found that, unsurprisingly, most journals assigned to taxonomically driven JCR categories (i.e., entomology and ornithology) were taxonomically specialised (Figure 1C). Meanwhile, taxonomically unrestricted journals dominated other JCR categories (Figure 1C).

### 3.2 Taxonomic Patterns Across Papers

We found that most papers in the field of ecology and evolution mention a single animal group (hereby *single‐animal‐group papers*) from the 10 categories we used for taxonomic classification (Figure S5A). However, the prevalence of single‐animal‐group papers in the literature has slightly decreased over the last 25 years (Figure S5A). We also observed that journals with higher JIF and JCI were more likely to publish papers (between 2021 and 2025) that did not specifically mention animals (e.g., conceptual or theoretical studies, empirical studies on humans, non‐animal organisms or the environment; Figure S5B,C). Although the distribution of both JIF and JCI was right‐skewed (Figure S5D,E), the patterns we found related to these metrics persisted when high‐impact journals were excluded from our analyses.

We gathered 991,894 ecology and evolution papers involving animals, the oldest of them published in 1877. 457,064 (46.1%) of these papers involved only invertebrates, 447,800 (45.1%) only vertebrates and 8730 (8.8%) both invertebrates and vertebrates. When partitioning animals into the 10 aforementioned groups, we observed that insects, mammals and fish were the most popular animals in this literature (Figure 2A). Still, insects were the least investigated animal group in relation to its diversity, while mammals and fish were the most overrepresented by the same standard (Figure 2B).

**FIGURE 2:**
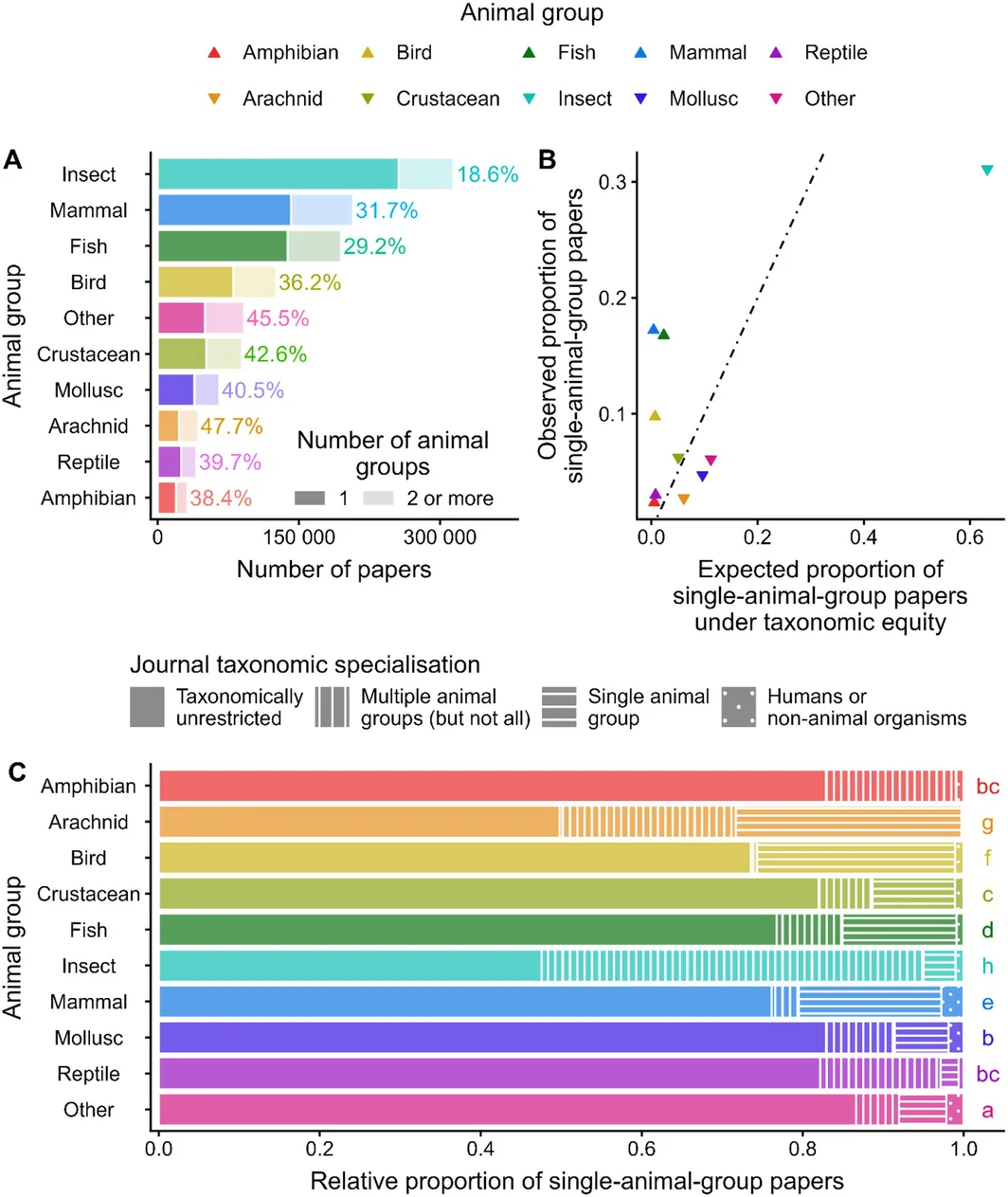
Taxonomic patterns across all papers mentioning at least one animal group. In Panel A, dark‐coloured bars represent the number of papers solely on that animal group, light‐coloured bars represent the number of papers examining that animal group in conjunction with other animals, and numbers beside these bars represent the percentages of papers examining that animal group in conjunction with other animals in relation to all papers on that animal group. Panel B shows the relationship between the expected proportion of single‐animal‐group papers under taxonomic equity (i.e., proportional to the species richness of animal groups according to Bánki et al. 2026) and the observed corresponding proportion, where the dot‐dashed line highlights a perfect relationship; points above the line signify overrepresentation, while points below the line signify underrepresentation. Panel C shows the relative proportion of single‐animal‐group papers on each animal taxon published in each type of journal taxonomic specialisation, in which distinct letters represent statistical differences between animal groups regarding their proportion of such papers in taxonomically unrestricted journals (*z*‐values with *p* < 0.05 for all pairwise comparisons).

Studies predominantly investigated each animal group in isolation, but the number of animal groups examined varied depending on the animals involved (Figure 2A). For instance, while 81.4% of papers involving insects focused solely on them, almost half (47.7%) of papers on arachnids concomitantly involved other animals (Figure 2A). This pattern could be even stronger when considering particular animals, such as ticks, a group of obligate parasites that represents 0.08% of all living arachnids (Bánki et al. 2026) but is mentioned by 20.1% of papers involving arachnids alongside other animals. We also noted that nearly half of all single‐animal‐group papers on insects and arachnids were published in taxonomically specialised journals (Figure 2C). Single‐animal‐group papers on other animals were mostly published in taxonomically unrestricted journals, although some more than others (Figure 2C).

We hereon focus on single‐animal‐group papers published in taxonomically unrestricted journals between 2001 and 2025 (*N*_papers_ = 560,364; *N*_journals_ = 375). Most of these papers focused on vertebrates overall (Figure 3A). However, when animals were divided into more specific groups, insects emerged as the single most frequently studied group, followed closely by fish, mammals and birds. Furthermore, when separating journals into five distinct sets (largely based on JCR categories), we noted that taxonomic patterns were not uniform across the literature of ecology and evolution. For instance, in 2025, approximately half of the papers published in aquatic‐related journals focused on fish, while most of the remaining papers examined invertebrates other than arachnids and insects (Figure 3B). By contrast, mammals and birds were particularly popular in journals focusing on behaviour, conservation and general ecology and evolution (Figure 3C–E), while zoology journals published mainly papers on invertebrates by 2025, primarily due to the high prevalence of papers on insects (Figure 3F). We additionally found that many taxonomically unrestricted journals showed extreme bias towards a single group of animals (Figure S6).

**FIGURE 3:**
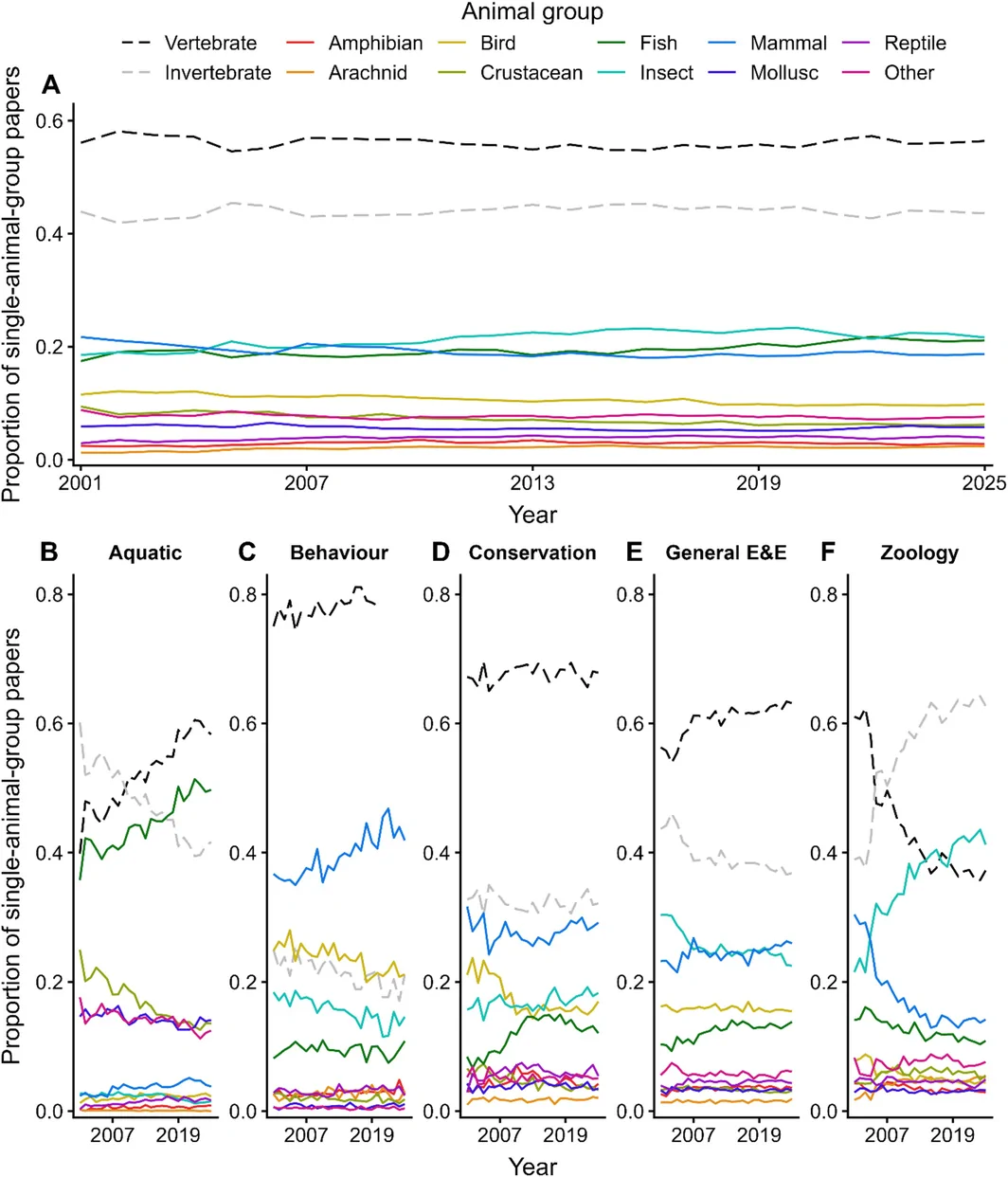
Proportion of single‐animal‐group papers investigating each animal group over time. While Panel A shows estimates for all journals together, Panels B–F show journals divided into five separate sets. Only papers published between 2001 and 2025 in taxonomically unrestricted journals were included in these analyses.

#### 3.2.1 In Relation to Time

The prevalence of papers on vertebrates remained the same over the last 25 years in the literature as a whole (Figure 3A) and in conservation journals (Figure 3D), yet the same could not be said for other journal sets. For instance, we observed that vertebrates became proportionally more studied than invertebrates in aquatic, behaviour and general ecology and evolution journals (Figure 3B,C,E). By contrast, vertebrates became proportionally less studied than invertebrates in zoology journals (Figure 3F).

Changes over time were more frequently observed when classifying animals into specific categories, as the prevalence of papers on 8 (out of 10) animal groups changed in the literature as a whole over the last 25 years (Figure 3A). However, these trends were small in magnitude: the strongest absolute change was the increase of 3.7% of papers on fish (Figure 3A). Temporal trends regarding taxa also occurred across various subfields, but often in distinct directions. For example, the proportion of papers on mammals increased in behaviour journals as well as in general ecology and evolution journals (Figure 3C,E) but remained constant in aquatic journals (Figure 3B) and decreased in conservation and zoology journals (Figure 3D,F). Importantly, some changes in taxonomic patterns over time within journal sets were enormous: fish accounted for 35.8% of papers published in aquatic journals in 2001 but 49.7% in 2025 (Figure 3B), while insects increased from 21.5% in 2001 to 41.3% in 2025 in zoology journals (Figure 3F).

#### 3.2.2 In Relation to Journal Impact Metrics

Papers published in 2021 to 2025 in journals with greater impact factor and citation indicator were more likely to focus on vertebrates (Figure 4). However, there were exceptions for this pattern: general ecology and evolution journals showed either a negative or an absent relationship between their impact metrics and prevalence of papers on vertebrates (Figure 4E,K), and behaviour journals strangely showed a positive trend between vertebrate pervasiveness in relation to JCI (Figure 4I) but a negative one for JIF (Figure 4C). Despite these trends, the prevalence of more specific animal groups in papers was rarely associated with journal impact metrics (Figure 4). For example, birds were as likely to be the focus of papers in high‐impact journals as in lower‐impact journals, regardless of journal set. We observed, however, a few trends related to taxon prevalence and journal impact metrics. For instance, the proportion of papers on insects across the entire literature was not associated with JIF but increased with JCI (Figure 4A,G). We observed the exact opposite pattern for papers on crustaceans, as their prevalence, both in the literature as a whole and across aquatic journals, decreased with JIF but not with JCI (Figure 4A,B). Meanwhile, taxonomic trends regarding JIF and JCI were congruent for fish and invertebrates other than arachnids, crustaceans, insects and molluscs in aquatic journals (positive and negative relationship, respectively; Figure 4G–J) and for mammals in journals of general ecology and evolution (negative relationship; Figure 4K,L).

**FIGURE 4:**
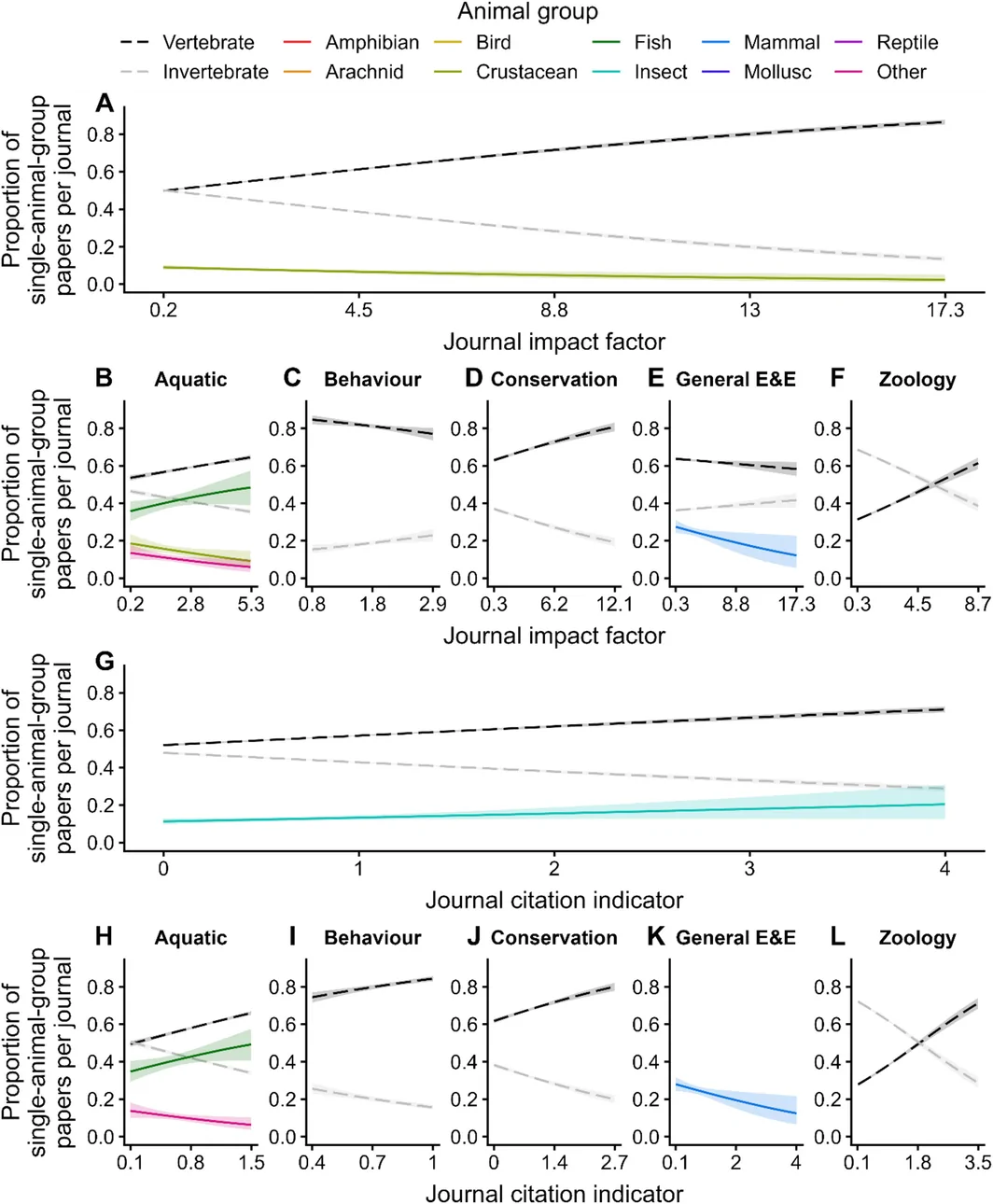
Proportion of single‐animal‐group papers investigating each animal group in relation to journal impact metrics. The journal impact metric explored in Panels A–F is 2024 journal impact factor, while 2024 journal citation indicator is explored in Panels G–L. Panels A and G show trends for all journals in ecology and evolution together, while Panels B–F and H–L do so within journal sets. Note that relationships for all taxa were analysed but only the ones that were consistently positive or negative (i.e., qualitative result remained the same when journal impact metric was restricted to lower values) are shown. Only papers published between 2021 and 2025 in taxonomically unrestricted journals were included in these analyses.

#### 3.2.3 In Relation to Individual Attention Received

Compared with papers on invertebrates within the same journal, papers on vertebrates in the ecology and evolution literature as a whole received, on average, 10.2% more citations (Figure 5A), 43.1% more online mentions (Figure 5B), 24.2% more readers in Mendeley (Figure 5C) and 45.1% higher Altmetric scores (Figure 5D). Our sensitivity analyses, which were restricted to a more complete (but much smaller) dataset from specific years, also showed that papers on vertebrates consistently received greater attention than papers on invertebrates within the same journal (Figure S7). Relative to our original analyses, the magnitude of these differences was similar for number of citations (9.3%–20%; Figure S7A–E), smaller for online mentions (12.7%–15.8%; Figure S7F–J) and greater for both Mendeley readers (31.9%–36.8%; Figure S7K–O) and Altmetric scores (29%–94.2%; Figure S7P–T).

**FIGURE 5:**
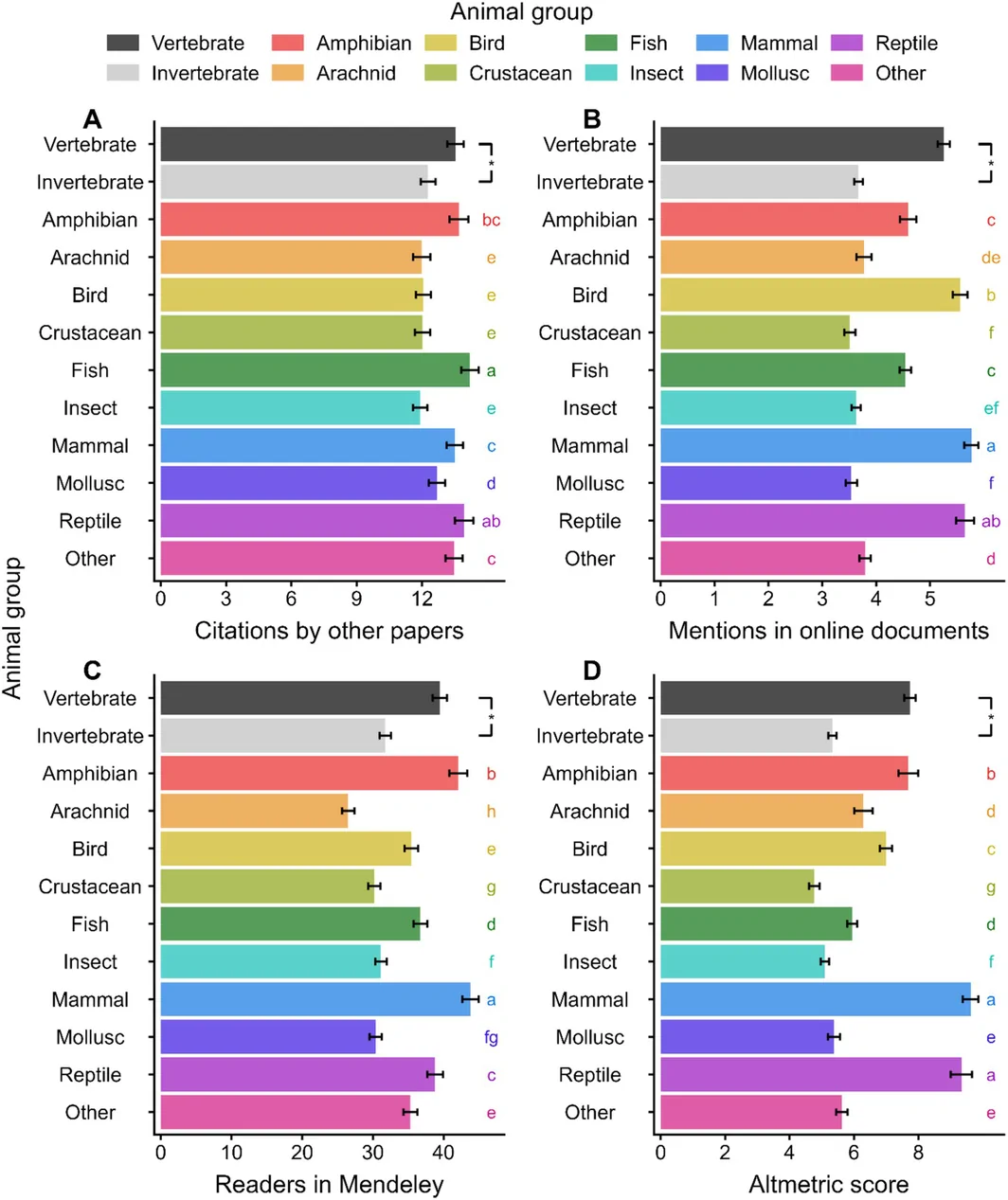
Attention metrics (panel A: number of citations, panel B: online mentions, panel C: Mendeley readers, panel D: Altmetric score) for papers on distinct animal groups within the same journal. Distinct letters represent statistical differences among papers on specific animal groups within each panel (*z*‐values with *p* < 0.05 for all pairwise comparisons), while differences between papers on vertebrates vs. invertebrates are shown with a star. Only papers published between 2001 and 2025 in taxonomically unrestricted journals were included in these analyses.

Within journal sets, we observed the same advantage of papers on vertebrates over papers on invertebrates across most attention metrics (Figure S8), except for citation counts in conservation journals, where no difference was detected (Figure S8C). Attention received by papers on different animal groups varied substantially across metrics and journal sets (Figure 5, Figures S7 and S8). For example, papers on birds often attracted the greatest online attention (Figure 5B; Figures S7G–J and S8F,H,I) but received the fewest citations (Figure 5A; Figures S7A–E and S8B–E).

## 4 Discussion

Our results show that ecology and evolution research is profoundly taxonomically unbalanced as certain animal groups are consistently neglected, both in representation (relative to diversity) in the literature and in the attention they receive. Although this imbalance has occurred for a long time, it has remained largely stable over the last 25 years when considering the ecology and evolution literature as a whole. Nonetheless, taxonomic patterns drastically changed within certain pockets in this field, with most of them showing an increasing prevalence of papers focusing on vertebrates over time (particularly fish in aquatic journals). Below, we discuss these patterns in detail, consider their implications for the study of ecology and evolution, and propose actions to ameliorate this issue.

We found that vertebrates are greatly overrepresented in the literature of ecology and evolution: they appear in 53.9% of all papers in this field mentioning animals despite representing only 4.7% of animal biodiversity (Bánki et al. 2026). In fact, the attention given to some invertebrates (e.g., ticks and other parasites) only occurs because of their association with vertebrates, a pattern that had already been noted by others (Simoncini et al. 2025). We thus corroborate and expand previous findings from specific pockets of ecology and evolution (Bonnet et al. 2002; Clark and May 2002; Rosenthal et al. 2017; Stahlschmidt 2011), revealing that taxonomic bias is a problem pertaining to the entire field and confirming an ‘institutional vertebratism’ (sensu Leather 2013). Nonetheless, we found substantial variation in taxonomic patterns across the ecology and evolution literature when animals were classified in more specific groups. For instance, research published in aquatic journals often involved fish, crustaceans, molluscs and other non‐arthropod invertebrates, while behaviour journals more commonly published research on mammals and birds. This variability indicates that there is not a single popular animal group across ecology and evolution. Furthermore, our discovery that species‐rich invertebrate groups (e.g., arachnids, insects) are often relegated to taxonomically specialised journals, which probably reach a narrower audience, suggests that the existing hyperfocus on vertebrates may be even more pronounced from the perspective of an average researcher.

The intense taxonomic bias favouring vertebrates in ecology and evolution has shown no signs of improvement since taxonomic bias was first discussed in the early 2000s. More specifically, we observed that the overrepresentation of vertebrates across the literature of ecology and evolution as a whole has remained stable or even increased in certain pockets of this field over the last 25 years. The only temporal increase in invertebrate‐focused studies during this period occurred in zoology journals, likely because these outlets concentrate much of (if not all) the existing research on systematics. This includes the revision of taxa and the description of new species, which are predominantly invertebrates precisely because of their immense and still largely undocumented diversity (Stork 2018), thus naturally inflating their representation within this journal set. Temporal trends regarding specific animal groups also varied across subdisciplines of ecology and evolution, suggesting that efforts to mitigate taxonomic bias will need to be tailored to the norms and priorities of each subfield. However, increasing research on invertebrates is undoubtedly critical to all subpockets of ecology and evolution.

We hypothesised that vertebrates, especially birds and mammals, would receive more attention than invertebrates across and within ecology and evolution journals. Indeed, compared with invertebrates, vertebrates were more likely to be the focus of papers published in journals with higher impact metrics. Likewise, papers on vertebrates acquired more citations, online coverage, Mendeley readers and greater Altmetric scores than papers on invertebrates within the same journal. Still, we note that these within‐journal results require extra caution because of the correlational nature of our dataset, uncontrolled variables (e.g., topic of papers) and incomplete (Altmetric) data. Our results are congruent with existing research on the attention given to distinct animal groups within and beyond academia. For instance, Rosenthal et al. (2017) found that studies on vertebrates were cited more frequently than those on arthropods. Moreover, vertebrates have been shown to receive more attention than invertebrates on social media (e.g., Instagram; Heathcote 2021), online knowledge platforms (e.g., Wikipedia; Mammola et al. 2023) and even everyday government‐curated imagery (e.g., postage stamps; Xie and Sun 2026). Nonetheless, using finer animal classifications showed more nuanced attention patterns: while journal impact metrics were rarely associated with the likelihood of publication for a specific animal group, the attention given to each animal group within a journal varied within and across subdisciplines and metrics.

It is possible that the attention patterns we identified within and across journals stem from researchers behaving differently depending on the organisms they study. For instance, ecologists and evolutionary biologists working on invertebrates may be more likely to submit their research manuscripts to taxonomically specialised or lower‐impact journals than those working with vertebrates. Researchers focusing on invertebrates may also struggle to contextualise their research into the existing theory, lowering their chances of publishing in impactful journals and receiving less attention overall. However, studies on invertebrates were previously found to cite vertebrates more often than studies on birds and mammals cited other taxa (Taborsky 2009), which suggests the opposite pattern (i.e., studies on vertebrates are less broadly contextualised than those on invertebrates). Thus, the taxonomic bias we identified in the ecology and evolution literature (and its persistence over time) probably occurs because of other processes.

While scientists' choice of research organisms can be influenced by many factors (Pollo and Kasumovic 2026), one of them is the perception that vertebrates are ‘better’ or more important than invertebrates, a view that is widespread in society (Miralles et al. 2019; Possidónio et al. 2019). This skewed valuation system, which is potentially used by gatekeepers (e.g., committees, journal editors, reviewers) to ascertain research appeal, may lead to taxonomically biased decisions. We argue that conducting science as a congeniality contest slows innovation (Broeckhoven and du Plessis 2022; Stuart‐Fox et al. 2023), curbs the generality of ecological and evolutionary knowledge (Bonnet et al. 2002; Dochtermann et al. 2026), underexposes society to animals that are ubiquitous in nature (Leather 2013), and inhibits the formation of academics exploring such animals (e.g., insect taxonomists; Gaston and May 1992; Leather 2009), further contributing to the problem in a vicious cycle. We thus believe that our results, including the attention advantage of vertebrates over invertebrates, should motivate changes within and outside of academia to reconsider how animals are valued, rather than serve as a pretext for individuals to justify discriminatory practices regarding taxa.

While several actions needed to reduce taxonomic bias involve the society at large (Pollo and Kasumovic 2026), we focus on possible interventions within academia to ameliorate this issue as they may be readily implemented. First, we urge publishers, journals and scientific societies to be more transparent about their decisions regarding publications and funding. This includes systematically collecting and publicly reporting information on the taxa investigated in submitted and accepted manuscripts, as well as in funded projects. This would be a relatively simple task by journals and funding institutions as authors or applicants can populate this information on submission. Second, taxonomic diversity should be explicitly incorporated into the guidelines for awards, grants, hiring and publication decisions, mitigating implicit biases among decision‐makers. Third, targeted financial and publishing incentives should be established to support researchers working on understudied taxa. Fourth, fomenting descriptive and fundamental research, such as taxonomy (Gaston and May 1992; Leather 2009) and natural history (Endler 2025; Rojas et al. 2026), is paramount to strengthen hypothesis‐driven and applied research, as one cannot test or apply undescribed organisms, processes and patterns. Although these propositions may be ambitious or even unrealistic, similar criticisms were once directed at affirmative action policies aimed at increasing the participation of underrepresented groups in academia. Many journals now state their commitment to diversity, equity and inclusion, yet none of those we examined explicitly promote research on neglected taxa. We hope that institutions will consider implementing our recommendations to foster a more taxonomically inclusive scientific community.

## Author Contributions

**Pietro Pollo:** conceptualization (lead), data curation (lead), formal analysis (lead), investigation (lead), methodology (lead), project administration (lead), validation (lead), visualization (lead), writing – original draft (lead), writing – review and editing (equal). **Michael M. Kasumovic:** conceptualization (supporting), writing – review and editing (equal).

## Funding

The authors have nothing to report.

## Conflicts of Interest

The authors declare no conflicts of interest.

## Supporting information


**Figure S1:** Clarivate's Journal Citation Report (JCR) classification of assessed journals. Numbers represent the number of journals within each set combination.
**Figure S2:** Taxonomic focus of papers published in journals specialised in one animal group. Panel A shows journals specialised in arachnids, birds, crustaceans or fish, while panel B shows remaining journals.
**Figure S3:** Taxonomic focus of papers published in journals specialised in multiple animal groups. Panel A shows journals that do not publish research on insects, while panel B shows journals that publish research on insects.
**Figure S4:** Altmetric data availability by publication year and 2024 journal impact factor. The shaded area represents the time window included in sensitivity analyses examining patterns in attention metrics.
**Figure S5:** Number of animal groups examined in papers in ecology and evolution. Panel A shows the proportion of papers on distinct numbers of animal taxa investigated from over the last 25 years (2001–2025). Panels B and C show the mean proportion of papers per journal on distinct numbers of animal taxa investigated collectively over the last 5 years (2021–2025) by 2024 journal impact factor and 2024 journal citation indicator, respectively. Panels D and E show the distribution of journal impact factor and journal citation indicator in 2024, respectively.
**Figure S6:** Most popular animal groups within journals. Bars represent the number of taxonomically unrestricted journals for which an animal group was the most frequently investigated. Dots represent the proportion of single‐animal‐group papers focusing on the most popular animal group in each journal, while squares represent the mean of these estimates per most popular animal group, with whiskers encompassing its 95% confidence interval. ‘Other invertebrate*’ refers to invertebrates other than arachnids, crustaceans, insects and molluscs.
**Figure S7:** Sensitivity analyses examining attention metrics patterns across taxa within journals. Distinct letters represent statistical differences among papers on specific animal groups within each panel (z‐values with *p* < 0.05 for all pairwise comparisons), while differences between papers on vertebrates vs. invertebrates are shown with a star. Only papers published in 2018 (A, F, K, P), 2019 (B, G, L, Q), 2020 (C, H, M, R), 2021 (D, I, N, S) or 2022 (E, J, O, T) in taxonomically unrestricted journals that had a 2024 impact factor up to 6 and published at least one paper focusing on each of the 10 animal groups evaluated were included in these analyses.
**Figure S8:** Attention metrics for papers across taxa within journals for distinct journal sets. Distinct letters represent statistical differences among papers on specific animal groups within each panel (z‐values with *p* < 0.05 for all pairwise comparisons), while differences between papers on vertebrates vs. invertebrates are shown with a star. Only papers published between 2001 and 2025 in taxonomically unrestricted journals were included in these analyses.
**Table S1:** Examples of how papers were manually classified regarding their taxonomic focus.
**Table S2:** Evaluation metrics for the algorithm used in our study to classify the taxonomic focus of studies in ecology and evolution. TP, TN, FP and FN stand for true positives, true negatives, false positives and false negatives, respectively.

## Data Availability

All data and code used and generated by this study, including keywords and model estimates, are available at https://zenodo.org/records/22759542.
