# Supplementary material for: Taxonomic Bias Persists in Ecology and Evolution Research Despite Decades of Concern
Source: Ecol Evol. 2026 Oct 4;16(10):e74450. doi: 10.1002/ece3.74450 (PMC13635473; doi:10.1002/ece3.74450)
Supplement: Supplementary file 1 [file ECE3-16-e74450-s001.docx]

# Supplementary material

## Supplementary figures

**Figure S1. Clarivate’s Journal Citation Report (JCR) classification of assessed journals.** Numbers represent the number of journals within each set combination.

**Figure S2. Taxonomic focus of papers published in journals specialised in one animal group.** Panel A shows journals specialised in arachnids, birds, crustaceans, or fish, while panel B shows remaining journals.

**Figure S3. Taxonomic focus of papers published in journals specialised in multiple animal groups.** Panel A shows journals that do not publish research on insects, while panel B shows journals that publish research on insects.

 **Figure S4. Altmetric data availability by publication year and 2024 journal impact factor.** The **s**haded area represents the time window included in sensitivity analyses examining patterns in attention metrics.

**Figure S5. Number of animal groups examined in papers in ecology and evolution.** Panel A shows the proportion of papers on distinct numbers of animal taxa investigated from over the last 25 years (2001-2025). Panels B and C show the mean proportion of papers per journal on distinct numbers of animal taxa investigated collectively over the last five years (2021-2025) by 2024 journal impact factor and 2024 journal citation indicator, respectively. Panels D and E show the distribution of journal impact factor and journal citation indicator in 2024, respectively.

**Figure S6. Most popular animal groups within journals.** Bars represent the number of taxonomically unrestricted journals for which an animal group was the most frequently investigated. Dots represent the proportion of single-animal-group papers focusing on the most popular animal group in each journal, while squares represent the mean of these estimates per most popular animal group, with whiskers encompassing its 95% confidence interval. “Other invertebrate*” refers to invertebrates other than arachnids, crustaceans, insects, and molluscs.

**Figure S7. Sensitivity analyses examining attention metrics patterns across taxa within journals.** Distinct letters represent statistical differences among papers on specific animal groups within each panel (z-values with p < 0.05 for all pairwise comparisons), while differences between papers on vertebrates *vs.* invertebrates are shown with a star. Only papers published in 2018 (A, F, K, P), 2019 (B, G, L, Q), 2020 (C, H, M, R), 2021 (D, I, N, S), or 2022 (E, J, O, T) in taxonomically unrestricted journals that had a 2024 impact factor up to 6 and published at least one paper focusing on each of the 10 animal groups evaluated were included in these analyses.

**Figure S8. Attention metrics for papers across taxa within journals for distinct journal sets.** Distinct letters represent statistical differences among papers on specific animal groups within each panel (z-values with p < 0.05 for all pairwise comparisons), while differences between papers on vertebrates *vs.* invertebrates are shown with a star. Only papers published between 2001 and 2025 in taxonomically unrestricted journals were included in these analyses.

## Supplementary tables

**Table S1. Examples of how papers were manually classified regarding their taxonomic focus.**

| **Details of specific papers** | **Focused animal taxa** | **Number of animal groups identified** |
| --- | --- | --- |
| Buchsbaum & Morse (2012) compared the behaviour of spiderlings released in the field with those fed in the laboratory with fruit flies. | Arachnids, insects | Two or more |
| Bochkov & O’Connor (2005) analysed the phylogeny and host associations of certain mites and murines. | Arachnids,  mammals | Two or more |
| Eraud et al. (2005)injected sheep red blood cells (SRBC, a common method to represent an immunological challenge) to understand the cost of immune responses on collared doves (*Streptopelia decaocto*). | Birds,  mammals | Two or more |
| Suzuki (2014) investigated the production of alarm calls in great tits (*Parus major*) by exposing their nests to three predator species (snakes, crows, and martens). | Birds, mammals, reptiles | Two or more |
| Tchana et al. (2025) assessed the effects of *Protocalliphora* larvae on the plumage colours of blue tits (*Cyanistes caeruleus*). | Birds, insects | Two or more |
| Milinski (1982) showed that three-spined sticklebacks prefer prey of familiar size by conducting experiments with water fleas (*Daphnia* *magna*) of various sizes as prey items. | Crustaceans,  Fish | Two or more |
| Coop et al. (2005) observed that black bears (*Ursus americanus*) forage on aggregations of cutworm moths (*Euxoa axiliaris*). | Insects,  mammals | Two or more |
| Cross & Jackson (2015) tested problem-solving skills of nine species of jumping spiders, mentioning that one of these species specialises on preying mosquitoes. | Arachnid | One |
| Latta et al. (2023) presented evidence of ivory-billed woodpeckers (*Campephilus principalis*) in Louisiana despite the US Fish and Wildlife Service suggesting that this species was extinct. | Birds | One |
| Mercado et al. (2005) explored song copying in humpback whales, mentioning in that song learning in birds is well understood. | Mammals | One |
| Iriarte et al. (1990) investigated the diet and behaviours of pumas (*Felis concolor*), finding that these animals feed from ungulates and compete with jaguars (*Panthera onca*). | Mammals | One |
| Olson et al. (2018) investigated patterns in the transcriptome of a tapeworm species (*Hymenolepis microstoma*). | Other invertebrate | One |
| García et al. (2008) assessed manure (of unmentioned animals) for management strategies. | - | Zero |

**Table S2. Evaluation metrics for the algorithm used in our study to classify the taxonomic focus of studies in ecology and evolution.** TP, TN, FP, and FN stand for true positives, true negatives, false positives, and false negatives, respectively.

| **Animal group** | **TP** | **TN** | **FP** | **FN** | **Total** | **Accuracy** | **Precision** | **Sensitivity** | **Specificity** | **F1 score** |
| --- | --- | --- | --- | --- | --- | --- | --- | --- | --- | --- |
| Amphibian | 49 | 1760 | 6 | 0 | 1815 | 0.997 | 0.891 | 1.000 | 0.997 | 0.942 |
| Arachnid | 55 | 1758 | 2 | 0 | 1815 | 0.999 | 0.965 | 1.000 | 0.999 | 0.982 |
| Bird | 194 | 1600 | 21 | 0 | 1815 | 0.988 | 0.902 | 1.000 | 0.987 | 0.949 |
| Crustacean | 107 | 1702 | 4 | 2 | 1815 | 0.997 | 0.964 | 0.982 | 0.998 | 0.973 |
| Fish | 204 | 1583 | 23 | 5 | 1815 | 0.985 | 0.899 | 0.976 | 0.986 | 0.936 |
| Insect | 407 | 1390 | 16 | 2 | 1815 | 0.990 | 0.962 | 0.995 | 0.989 | 0.978 |
| Mammal | 260 | 1505 | 48 | 2 | 1815 | 0.972 | 0.844 | 0.992 | 0.969 | 0.912 |
| Mollusc | 82 | 1730 | 3 | 0 | 1815 | 0.998 | 0.965 | 1.000 | 0.998 | 0.982 |
| Reptile | 62 | 1742 | 8 | 3 | 1815 | 0.994 | 0.886 | 0.954 | 0.995 | 0.919 |
| Other | 126 | 1681 | 6 | 2 | 1815 | 0.996 | 0.955 | 0.984 | 0.996 | 0.969 |

## References

Bochkov, A. V., and B. M. OConnor. 2005. “Phylogeny and Host Associations of the Fur-Mite Subgenus *Listrophoroides* (*Sensu stricto*) Hirst (Acari : Atopomelidae) With an Intriguing Example of Synhospitality on Rats of the Genus *Maxomys*.” *Invertebrate Systematics* 19, no. 5: 437-498. <https://doi.org/10.1071/IS05017>

Buchsbaum, D., and D. H. Morse. 2012. “The Effect of Experience and Rearing Environment on the Behaviour of Crab Spiderlings During Their First Weeks of Life. *Behaviour* 149, no. 6: 667-683. <https://doi.org/10.1163/156853912X649939>

Coop, J. D., C. D. Hibner, A. J. Miller, and G. H. Clark. 2005. “Black Bears Forage on Army Cutworm Moth Aggregations in the Jemez Mountains, New Mexico”. *Southwestern Naturalist* 50, no. 2: 278-281. [https://doi.org/10.1894/0038-4909(2005)050[0278:BBFOAC]2.0.CO;2](https://doi.org/10.1894/0038-4909(2005)050%5b0278:BBFOAC%5d2.0.CO;2)

Cross, F. R., and R. R. Jackson. 2015. “Solving a Novel Confinement Problem by Spartaeine Salticids That Are Predisposed to Solve Problems in the Context of Predation”. *Animal Cognition* 18, no. 2: 509-515. <https://doi.org/10.1007/s10071-014-0819-z>

Eraud, C., O. Duriez, O. Chastel, and B. Faivre. 2005. “The Energetic Cost of Humoral Immunity in the Collared Dove, *Streptopelia Decaocto*: Is the Magnitude Sufficient to Force Energy‐Based Trade‐Offs?” *Functional Ecology* 19, no. 1: 110-118. <https://doi.org/10.1111/j.0269-8463.2005.00934.x>

García, A. M., T. L. Veith, P. J. A. Kleinman, C. A. Rotz, and L. S. Saporito. 2008. “Assessing Manure Management Strategies Through Small-Plot Research and Wholefarm Modeling.” *Journal of Soil and Water Conservation* 63, no. 4: 204-211. <https://doi.org/10.2489/jswc.63.4.204>

Iriarte, J. A., W. L. Franklin, W. E. Johnson, and K. H. Redford. 1990. “Biogeographic Variation of Food Habits and Body Size of the America Puma.” *Oecologia* 85, no. 2: 185-190. <https://doi.org/10.1007/BF00319400>

Latta, S. C., M. A. Michaels, T. C. Michot, P. L. Shrum, P. Johnson, J. Tischendorf, M. Weeks, et al. 2023. “Multiple Lines of Evidence Suggest the Persistence of the Ivory‐Billed Woodpecker (*Campephilus principalis*) in Louisiana.” *Ecology and Evolution* 13, no. 5: e10017. <https://doi.org/10.1002/ece3.10017>

Mercado, E., L. M. Herman, and A. A. Pack. 2005. “Song Copying by Humpback Whales: Themes and Variations.” *Animal Cognition* 8, no. 2: 93-102. <https://doi.org/10.1007/s10071-004-0238-7>

Milinski, M. 1982. “Optimal Foraging: The Influence of Intraspecific Competition on Diet Selection.” *Behavioral Ecology and Sociobiology* 11, no. 2: 109-115. <https://doi.org/10.1007/BF00300099>

Olson, P. D., M. Zarowiecki, K. James, et al. 2018. “Genome-Wide Transcriptome Profiling and Spatial Expression Analyses Identify Signals and Switches of Development in Tapeworms.” *EvoDevo* 9, no. 1: 21. <https://doi.org/10.1186/s13227-018-0110-5>

Suzuki, T. N. 2014. “Communication About Predator Type by a Bird Using Discrete, Graded and Combinatorial Variation in Alarm Calls.” *Animal Behaviour* 87: 59-65. <https://doi.org/10.1016/j.anbehav.2013.10.009>

Tchana, C. N., A. Fargevieille, L. Sandmeyer, et al. 2025. “*Protocalliphora* Larvae: Moderate but Lasting Carry-Over Effects on Yearling and Mother Coloration.” *Behavioral Ecology* 36, no. 4: araf044. <https://doi.org/10.1093/beheco/araf044>
